# DNA-Binding Activities of KSHV DNA Polymerase Processivity Factor (PF-8) Complexes

**DOI:** 10.3390/v17020190

**Published:** 2025-01-29

**Authors:** Jennifer Kneas Travis, Megan Martin, Lindsey M. Costantini

**Affiliations:** 1 Department of Biological and Biomedical Sciences, North Carolina Central University, Durham, NC 27707, USA; jkneas@eagles.nccu.edu (J.K.T.); mmarti64@nccu.edu (M.M.); 2 Integrated Biosciences (INBS) Doctoral Program, North Carolina Central University, Durham, NC 27707, USA

**Keywords:** KSHV, HHV-8, viral replication, electron microscopy, human herpesviruses

## Abstract

Kaposi’s Sarcoma Herpesvirus (KSHV) is the causative agent of several human diseases. There are few effective treatments available to treat infection and KSHV oncogenesis. Disrupting the KSHV infectious cycle would diminish the viral spread. The KSHV lytic phase and production of new virions require efficient copying and packaging of the KSHV genome. KSHV encodes its own lytic DNA replication machinery, including the processivity factor (PF-8), which presents itself as an attractive target for antiviral development. We characterized PF-8 at the single molecule level using transmission electron microscopy to identify key molecular interactions that mediate viral DNA replication initiation. Our results indicate that PF-8 forms oligomeric ring structures (tetramer, hexamer, and/or dodecamer) similar to the related Epstein–Barr virus processivity factor (BMRF1). Our DNA positional mapping revealed high-frequency binding locations of PF-8 within the lytic origin of replication (OriLyt). A multi-variable analysis of PF-8 DNA-binding activity with three mutant OriLyts provides new insights into the mechanisms that PF-8 associates with viral DNA and complexes to form multi-ring-like structures. Collectively, these data enhance the mechanistic understanding of the molecular interactions (protein–protein and protein-DNA) of an essential KSHV DNA replication protein.

## 1. Introduction

Kaposi’s Sarcoma Herpesvirus (KSHV) is a part of the gamma subfamily of the Herpesviridae family along with Epstein–Barr Virus (EBV) [1]. The Herpesviridae family consists of large linear double-stranded DNA genomes that are encompassed by an icosahedral capsid and consist of an envelope that has many viral glycoproteins [2,3]. KSHV is the causative agent of Kaposi’s sarcoma (KS), multicentric Castleman’s disease, primary effusion lymphoma, and KSHV inflammatory cytokine syndrome [3,4,5,6]. KSHV is most prevalent in African and Mediterranean regions [7]. The mode of transmission may vary in different regions and KSHV can infect a broad range of cell types including endothelial cells, epithelial cells, B-cells, monocytes, and keratinocytes [3,7]. There are few effective treatments available for KSHV because of several difficulties such as side effects and limited efficacy; however, there are ongoing efforts to discover antivirals that target the KSHV lifecycle [8,9].

KSHV like all herpesviruses exists in a biphasic state: latent (dormant) and lytic (active) [10]. Both phases contribute to oncogenesis through various mechanisms such as proliferation, angiogenesis, and inflammation [11]. The lytic phase is when new virions are produced and more specifically this is when the viral genome is replicated by a set of lytic DNA replication proteins that are encoded by KSHV [12]. The lytic DNA replication proteins are encoded by ORF50 (origin binding protein/RTA), ORF9 (DNA polymerase/POL), ORF44 (helicase), ORF6 (single-stranded binding protein), ORF40/41 (primase associated factor), ORF56 (primase), and ORF59 (processivity factor/PF-8) [12]. Some of these proteins are known to associate with one another and form subcomplexes [13]. In addition to the seven crucial lytic DNA replication proteins, there are two lytic origins of replication (OriLyt). Previous studies have identified the motifs shared between the two sequences, which are an AT-rich region, AT-palindrome, TATA boxes, RTA responsive element (RRE) site, and AP-1 and CAAT binding sequences [14,15]. The OriLyts also share a GC-rich repetitious region [14]. Currently, there are two proposed mechanisms for how the pre-initiation DNA replication complex forms. One mechanism proposes that the KSHV DNA replication proteins associate together in solution and subsequently bind to the OriLyt. The second mechanism proposes that the origin binding proteins (RTA and K8) associate with the OriLyt and recruit other proteins to the area to form the pre-initiation complex on DNA [16]. In addition to viral proteins, cellular proteins such as topoisomerases, RecQL, and Ku86/70 autoantigens are important for the formation of the pre-initiation complex [16].

Processivity factors are one of many proteins involved in forming the pre-initiation complex and are required for the efficient replication of the viral genome. The main function of processivity factors is to help keep DNA polymerase bound to DNA so that the 165-170 kilobase KSHV genome can be synthesized efficiently [17,18]. Without PF-8, the DNA polymerase is only capable of incorporating three nucleotides before DNA polymerase dissociates from the DNA further emphasizing the importance that PF-8 plays in lytic DNA replication [17]. PF-8 encodes several functional domains including dimerization, double-stranded DNA binding, DNA polymerase binding, and a nuclear localization signal [19]. Each of those domains serves a critical role in lytic DNA replication [20]. The crystal structure of a truncated PF-8 isolated from an *E. coli* expression system revealed the formation of dimers by head-to-head association, while a study with cell lysate from reactivated iSLK BAC 16 cells suggests that PF-8 is capable of forming a tetramer [19,21]. There are several outstanding questions we seek to investigate using our imaging approach.

The goal of this study is to characterize purified full-length PF-8 at the single molecule level using transmission electron microscopy (TEM). We have successfully applied similar approaches to show RTA binds to specific DNA sequences as a dimer and throughout the OriLyt as a monomer [22]. The advantage of utilizing electron microscopy is that it allows for heterogeneous populations to be observed. We utilized two different electron microscopy approaches: negative staining to observe protein conformations and tungsten rotary shadowing to analyze PF-8’s position on OriLyt DNA and protein area in the presence and absence of DNA [23,24]. We characterized the oligomeric state of PF-8 in the presence and absence of viral DNA. Our data suggest that PF-8 can form multiple oligomeric states with some complexes larger than a tetramer. We identified several PF-8 binding locations within the OriLyt and generated three mutant OriLyt DNAs to further characterize PF-8’s preferential binding. PF-8 has been previously shown to associate with the CAAT and RRE regions within the OriLyt [25,26]. Our data provides evidence that PF-8 can preferentially bind to regions encoding CAAT sites; however, our mutant DNA templates suggest a DNA structural specificity as well. Similar to our findings with RTA, we observed DNA-binding preferences for the different PF-8 oligomers. By helping provide a comprehensive characterization of PF-8 at the single molecule level, we hope to provide a better understanding of PF-8’s role in DNA replication and thus provide a more targeted approach for the development of antivirals against KSHV.

## 2. Materials and Methods

### 2.1. Biotin End-Labeling of DNA

The OriLyt was synthesized from GeneArt (ThermoFisher, Waltham, MA, USA) and was subcloned into either pRSET or poxGFP-N1 [22]. Approximately 80 units of XhoI (New England Biolabs, Ipswich, MA, USA) was digested for 1 h at 37 °C with the OriLyt to create a 5′ overhang. The restriction enzyme was then heat-inactivated, and 20 units of Klenow Exo-Polymerase (New England Biolabs), 2.8 µM of biotin 14-CTP, and 37µM of dATP, dGTP, and dTTP were added and incubated for 1 h at 37 °C. Subsequently, the polymerase was heat-inactivated, and either 80 units of NdeI (OriLyt-R poxGFP-N1) or NdeI and ScaI (OriLyt-R pRSET) were added and incubated at 37 °C for 1 h. The samples were then run on a 0.5 or 1.0% agarose gel cast with GelRed Nucleic Acid Stain (Biotium, Fremont, CA, USA), and the approximate 2.4kb (OriLyt-R) band was excised and purified with the QIAquick Gel Extraction Kit (Qiagen, Hilden, Germany) following manufacturer’s instructions. DNA concentrations were determined using the Nanodrop 2000c spectrophotometer (ThermoScientific, Waltham, MA, USA).

### 2.2. Site-Directed Mutagenesis of Lytic Origins of Replication

To introduce mutations, a shuttle vector was engineered for the OriLyt that used restriction sites PstI and XhoI, flanking a 1.28 kb region of the OriLyt that was subcloned into poxGFP-N1. Mutagenesis was performed in the shuttle vector. The mutagenesis primers used are listed in Supplementary Table S1 and were designed to mutate certain CAAT sites or a TATA box within the OriLyt. Site-directed mutagenesis was performed with Pfu polymerase according to the manufacturer’s instructions (Agilent Technologies, Santa Clara, CA, USA). Following site-directed mutagenesis PCR, the PCR reaction was incubated with DpnI (New England Biolabs) for 1 h at 37 °C. Subsequently, PCR products were purified using the QIAquick PCR Purification Kit (Qiagen) and transformed into DH5α component cells (ThermoFisher) following the manufacturer’s instructions. Several clones were cultured, and the DNA was isolated with the GeneJET Plasmid Miniprep Kit (ThermoFisher). Once mutations were sequence verified, the positive clones were transformed with standard cloning techniques, subcultured, and DNA was isolated with the Qiagen Plasmid Midi Kit (Qiagen). The sequence-verified mutated DNA was subcloned back into the full-length OriLyt vector and sequenced to confirm successful cloning. Two triple-mutants (mutant 1 and 2) and one six-point mutant (mutant 3) were generated using the outlined methods.

### 2.3. Denaturing and Semi-Native Gel Electrophoresis

PF-8 was expressed and purified from Sf9 insect cells (ThermoFisher). Briefly, Sf9 cells were harvested 48 h post-infection and PF-8 was isolated using a HisTrap (Waltham, MA, USA) column with a linear gradient (20–500 mM imidazole in 25 mM Tris, 500 mM NaCl). The His-tag was cleaved using a TEV protease, and the TEV protease was removed using Dynabeads (Waltham, MA, USA). PF-8 was then dialyzed against PBS for 16 h. Purified PF-8 was aliquoted and flash-frozen using liquid nitrogen and stored at −80 °C. Glutamine synthetase (GS) was expressed and purified from *E. coli* (Sigma Aldrich, St. Louis, MO, USA). GS was reconstituted to a concentration of 608 ng/µL according to the manufacturer’s recommendations in water, aliquoted, flash frozen, and stored at −80 °C. Approximately 4.0 µg of PF-8 or GS was added to 1× Laemmli sample buffer with 2.5% BME (Biorad, Hercules, CA, USA) or 1× semi-native loading buffer (31.3 mM Tris, 0.005% bromo blue, 0.1% SDS, and 20% glycerol). Samples that contained Laemmli sample buffer were incubated at 95 °C for 10 min and vortexed. For the semi-native gel, a 4–20% Mini-Protean TGX Stain-free protein gel (Biorad) was pre-run at 70 V for 20 min with 1× TG buffer on ice. Samples and ladder (NativeMark unstained protein standard, Invitrogen (Waltham, MA, USA)) were loaded into the gel and run for 20 min at 70 V and then for 100 V for 4 to 5 h. For the denaturing gel, the samples and ladder (Precision Plus Protein Kaleidoscope, Biorad) were loaded into a 4–20% Mini-Protean TGX Stain-free protein gel (Biorad) and run for 100 V until the dye front was at the bottom of the gel. To stain the polyacrylamide gels, the gels were microwaved for 1 min in deionized water twice with a water exchange and then microwaved for 30 s with Coomassie stain (0.05% R-250, 50% methanol, and 10% acetic acid). The gels were incubated with the Coomassie stain for 15 min at room temperature and were destained with deionized water overnight. Images of the gels were taken using the Chemidoc MP Imaging system (Biorad).

### 2.4. Negative Staining of PF-8

For negative staining, PF-8 was diluted in 20 mM HEPES and 150 mM NaCl to concentrations of 1–30 ng/µL. Carbon-coated 400-cooper mesh grids were glow-discharged for 2 min at 150–200 torr. Next, 15 µL of PF-8 protein was absorbed onto the glow-discharged grids for 5 min. The grid was stained with 1–2% (*w*/*v*) uranyl acetate diluted in water and dried on filter paper. TEM images were obtained using a FEI Tecnai 12 electron microscope at 80 kV with 26,000–60,000× magnification and captured on a Gatan First Light CCD camera using Gatan Digital Micrograph software version 1.85.1535 (Gatan, Pleasanton, CA, USA).

### 2.5. Tungsten Rotary Shadowing of Purified Proteins and DNAs

For the condition of PF-8 at 4 °C, PF-8 was diluted to a final concentration of 2 ng/µL in EM buffer (20 mM HEPES, 50 mM NaCl, 0.1 mM EDTA, 0.5 mM DTT) and incubated on ice for at least 2 h. For all other PF-8 conditions, PF-8 was diluted in EM buffer to a final concentration of 54.8 ng/µL. For increasing incubation time, 200 ng of PF-8 was incubated with EM buffer for 30 min, 1 h, 2 h, or 3 h at room temperature and then absorbed onto glow-discharged, carbon-coated copper mesh grids. For increasing concentrations 100 ng, 200 ng, 400 ng, or 800 ng of PF-8 was combined with EM buffer and incubated for 1 h at room temperature before being absorbed onto the glow-discharged, carbon-coated copper mesh grids. GS was diluted in water to a final concentration of 101.3 ng/µL and incubated on ice for 1 h. Subsequently, 200 ng of GS was absorbed onto glow-discharged, carbon-coated copper mesh grids.

For DNA-binding reactions, 200 ng of biotin-labeled DNA and EM buffer were incubated at room temperature for 10 min. PF-8 was diluted in EM buffer to a final concentration of 43.2 ng/µL. Then, 1080 ng of PF-8 was added to the DNA reaction and incubated at room temperature for 30 min. For fixed samples, 0.3% glutaraldehyde was added next, incubated for 15 min at room temperature, and quenched with 0.1 mM Tris. In order to label the DNA, 0.01 mg/mL of streptavidin was added and incubated for at least 15 min at room temperature. A size exclusion column was prepared with 6% agarose beads (Agarose Bead Technologies, Torrejón de Ardoz, Spain) and equilibrated with TE (10mM Tris-base (pH 7.6), 1mM EDTA with LC/MS water) for 30 min. The DNA–protein reaction was then added to the column and the void volume for the column was collected and discarded. Subsequently, fractions (2 drops) were collected and measured using Qubit dsDNA HS Assay Kit (ThermoFisher) following the manufacturer’s instructions to identify fractions to be mounted for TEM.

Carbon-coated 400-cooper mesh grids (Electron Microscopy Sciences, EMS, Hatfield, PA, USA) grids were glow-discharged for 45 s. Fractions from the size exclusion column that contained the highest DNA concentration according to Qubit were mixed with 2 mM spermidine and absorbed onto the glow-discharged grids for 5 min. The grids were then quickly rinsed in a dish containing ultrapure LC/MS water and placed in another dish of LC/MS water for 3 min. Subsequently, the grid was placed in increasing concentrations of ethanol (25%, 50%, 75%, and 100%) for 5 min each. The grids were then either shadowed the same day or stored at room temperature until shadowing could be performed. For shadowing, the grids were shadowed with evaporated tungsten until the deposition monitor reached 0.65 kÅ. The grids were then immediately imaged or stored in a vacuum desiccator (Pelco 2251 Vacuum Desiccator) until imaging the next day. Imaging was performed with the Philips CM12 TEM microscope with Gatan Orius charged couple camera and Gatan Digital Micrograph Software was used to acquire images. The TEM microscope was set to 40 kV and 13,000× magnification for samples shadowed with tungsten.

### 2.6. Quantification of DNA Structures

Grids that contained wild-type or mutant 1–3 OriLyt DNA with PF-8 were imaged using a Philips CM12 TEM microscope, and roughly 400 DNA molecules per replicate were counted by choosing multiple fields of view on the grid and quantifying the different DNA and protein conformations. For each experiment, two grids were examined for each replicate of the wild-type and mutant OriLyts.

### 2.7. Single Molecule Analysis of PF-8 Position and Area

Electron micrographs were converted from dm3 files to tiff files and analyzed for protein area and position of PF-8 using a Macro for ImageJ (NIH). Protein area was measured in pixels^2^ using the elliptical selection tool and converted to nm^2^. The oligomeric states of PF-8 were subdivided into three different categories: <200 nm^2^ (monomer/dimer), 200–325 nm^2^ (hexamer/dodecamer), and >325 nm^2^ (>dodecamer). The cut-offs for the different oligomeric states were determined by looking at the distribution and/or groupings of the dot plots for PF-8′s area under the different conditions while also considering the distribution pattern for GS (molecular standard).

The DNA position of PF-8 was determined by measuring the DNA length from the end of streptavidin to the beginning of PF-8 in pixels then that value was converted to nanometers and subsequently to base pairs. The conversion factor from pixels to nanometers was encoded in the dm3 files. The conversion factor of 2.9 bp/nm is based on the known distance between base pairs and nanometers [27]. The frequency histograms have bin sizes set to 75 bp. Ultimately, 75 bp was chosen based on the diameter of PF-8 that was calculated from the area of PF-8, assuming a circular shape. Percentages of the quantification of different DNA and protein conformations were determined by totaling each conformation/phenotype from the three replicates and dividing it by the total number of DNA molecules counted in the three replicates and then multiplying by 100 for each DNA (OriLyt and mutant 1–3). The calculated bend angles for induced protein binding to DNA were determined by using the angle tool in ImageJ to measure the opening angle and then subtracting that value from 180. The lines for measuring the opening angle were positioned so that they were approximately tangent to the DNA on opposite sides of the protein [28]. The DNA bending angle was not calculated if the protein bound to the end of DNA or if protein binding induced a loop in the DNA. The protein area for the heat maps was subdivided into three groups: >325 nm^2^, 200–325 nm^2^, and <200 nm^2^ while the DNA sequence was grouped in bins of 75 bp.

### 2.8. Data Analysis and Statistics

Dot plots, frequency histograms, bar graphs, and heat maps were generated using GraphPad (Prism). Kruskal–Wallis with Dunn’s multiple comparison test was used to determine statistical significance for protein area, Supplementary Table S2. Images were compiled and annotated using Adobe Photoshop and Illustrator. Sequence-specific analysis for the OriLyt was performed with CLC Main Workbench (Qiagen). Models were generated using BioRender. Truncated PF-8 structures (PDB: 3HSL) were modeled with ChimeraX [21,29].

## 3. Results

### 3.1. Purified PF-8 Forms Ring Structures In Vitro

**Figure 1:**
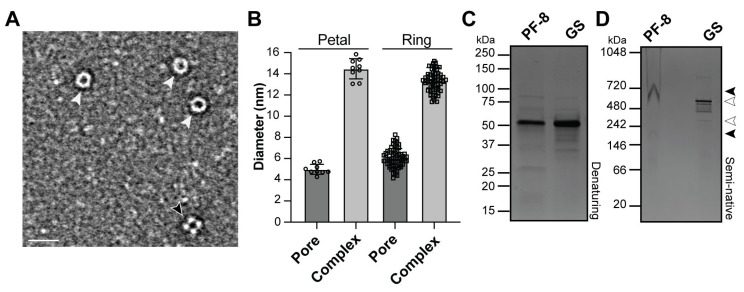
Detection of higher molecular weight PF-8 oligomers: (**A**). Representative electron micrograph of PF-8 using negative staining revealed two distinct conformations. White arrowheads denote ring conformation and black arrowhead denote a petal conformation. Scale bar = 25 nm (**B**). Quantification of the diameter of the central pore and complex of the petal and ring conformations was carried out by manually measuring. Petal pore: 5.00 ± 0.47 nm, petal complex: 14.48 ± 0.95 nm (n = 9), ring pore: 6.07 ± 0.89 nm, ring complex: 13.29 ± 0.96 nm (n = 64). (**C**). Denaturing gel of PF-8 (52 kDa) and GS (51.9 kDa). (**D**). Semi-native gel of PF-8 (filled arrowheads) and GS (open arrowheads). PF-8 forms a possible dodecamer (52 kDa × 12 = 624 kDa) and tetramer (52 kDa × 4 = 208 kDa). GS possibly forms dodecamer (622.8 kDa) and hexamer (311.4 kDa).

DNA processivity factors are known to form a range of protein complexes [1,30]. For example, the eukaryotic sliding clamp forms a trimer, Herpesvirus simplex-1 forms a monomer, and EBV forms a dimer, tetramer, and possible larger complexes [1,30,31,32,33]. With the differences in multimeric states observed among processivity factors, we sought to investigate the structure(s) PF-8 forms with a single molecule EM analysis and gel-based approaches. Using an imaging approach, we observed that PF-8 forms two distinct conformations: a petal-shaped structure that we hypothesize to be a tetramer and a ring-shaped structure we predict to be a hexamer (Figure 1A). In addition, both conformations contain a central pore (petal: 5.0 ± 0.47 nm and ring: 6.1 ± 0.89 nm) that is large enough to accommodate the width of the double helix of DNA; therefore, we hypothesize that PF-8 has the ability to encircle DNA (Figure 1B) [34]. Gel analysis revealed a PF-8 band at approximately 52 kDa under denaturing conditions, which correlates with previous studies (Figure 1C) [19,35]. Based on the amino acid sequence of PF-8, the predicted monomeric weight is 42.4 kDa; therefore, the observed increase in molecular weight is likely due to protein post-translational modifications [35,36]. Glutamine synthetase (GS) which we used as a protein standard forms a ring-shaped structure with a central pore and has a similar monomeric weight (51.9 kDa) as PF-8 [37,38]. Next, we used a semi-native gel that contained a low amount of SDS to resolve the native folded state(s) of PF-8 and GS [39]. The semi-native gel revealed a band present for GS around the size of the dodecamer, 12-mer (622.8 kDa), and a faint band around the predicted size of a hexamer (311.4 kDa, Figure 1D). GS in other species are known to form several oligomeric states; therefore, perhaps we are detecting less common oligomeric states that correspond to the bands present between the dodecamer and hexamer [40]. Alternatively, bands in a native gel migrate not only based on size but also shape; therefore, it is a possibility that we are observing different protein orientations present in the gel [41]. We consistently observed the PF-8 bands appeared as a W-shaped band, likely because PF-8 has a high pI value (8.9), which will impact the ability of the protein to enter and efficiently migrate in the gel [42]. Nonetheless, the prominent band (between 480–720 kDa) is comparable to the size of the GS dodecamer, and there is a faint band (between 146–242 kDa) detected around the predicted size of a tetramer (denaturing gel PF-8 band molecular weight 52 kDa, 52 × 4 = 208 kDa). The results from the semi-native gel suggest that the ring-shaped structure present in the negative staining data could be two hexameric rings stacking on top of each other forming a dodecamer. Furthermore, the semi-native gel results indicate that the dodecamer band is present in greater abundance than the tetramer based on band intensity, which correlates with the ring-shaped structure (predicted dodecamer, n = 64), with negative staining being more frequently observed than the petal structure (predicted tetramer, n = 9). These results suggest that purified, full-length PF-8 can form a tetramer, hexamer, and/or dodecamer in the absence of DNA.

**Figure 2:**
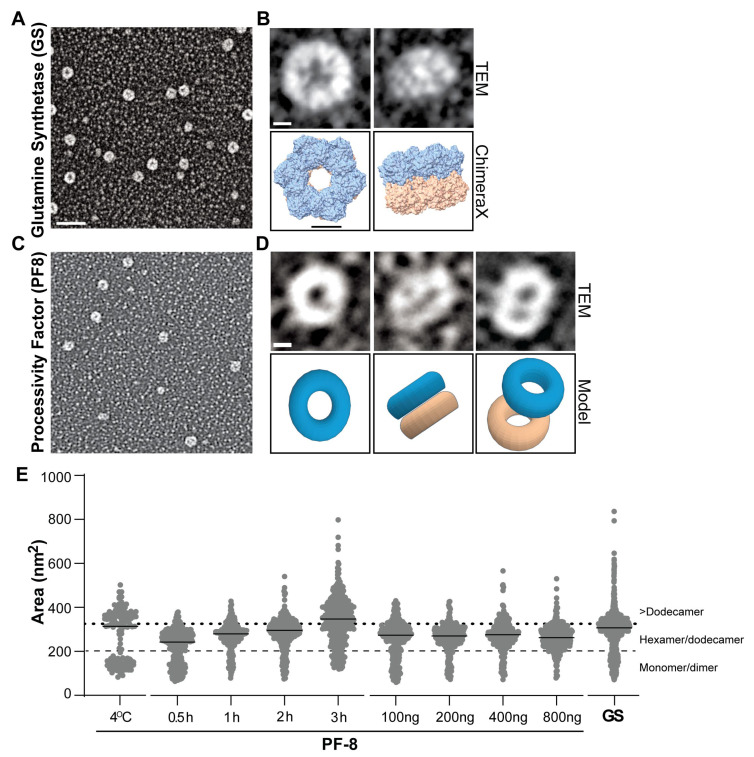
Single-molecule EM analysis of GS and PF-8 areas: (**A**). Representative electron micrograph of GS with tungsten rotary shadowing. Scale bar = 50 nm. (**B**). Single-molecule comparison of individual molecules of GS observed with matched known structures (PDB: 8PVG). Scale bar = 5 nm. (**C**). Representative electron micrograph of PF-8 with tungsten rotary shadowing. (**D**). Single-molecule analysis of PF-8 with a proposed model representation of the observed structures. Scale bar = 5 nm (**E**). Dot plots of the area of PF-8 over a range of conditions: 4 °C, 0.5−3 h (200 ng at room temperature), 100−800 ng (1 h incubation at room temperature), and GS. The median values are shown for each condition by the horizontal line. Dotted and dashed lines represent cutoffs for the different PF-8 oligomers: monomer/dimer, hexamer/dodecamer, and >dodecamer.

Next, we went on to explore how time and concentration influence the dynamics of PF-8′s protein–protein interactions involved in assembling higher oligomeric states. GS and PF-8 under varying times, concentrations, and temperatures were mounted onto glow-discharged, carbon-coated copper mesh grids, shadowed with evaporated tungsten, imaged, and analyzed. The electron micrographs revealed that GS predominantly forms a ring-like conformation with a central pore (Figure 2A). When comparing the individual GS molecules in the micrographs to the known structure (PDB: 8PVG), GS primarily exhibits a top-down view that is present the majority of the time, and a side view that is less prevalent (Figure 2B) [43]. Previously, the top-down view was the most frequent conformation when GS was stained with uranyl acetate [40]. PF-8 at the different incubation times mostly forms an annulus or ring-shaped conformation (Figure 2C). Figure 2D includes an electron micrograph and paired models of two ring-shaped structures assembling to form the dodecamer. Similar to GS, PF-8 forms a ring-shaped structure with a central pore and can display a similar side view. In addition, the formation of the hypothesized dodecamer state can be observed with two ring-shaped structures off-center from one another.

We compared the diameters and areas of GS and PF-8 (Supplementary Figure S1). Assuming a circular shape we used the measured area to calculate the diameters of PF-8 and GS prepared using tungsten rotary shadowing (Supplementary Figure S1A,B). The diameters of PF-8 and GS were 19.5 nm and 20.5 nm, respectively. The comparable diameter and area support PF-8 forming stacked hexamers (dodecamer). To approximate the number of monomers per ring structure visualized using negative staining, the area of the ring excluding the central DNA pore was divided by the measured area of a monomer (assuming a subunit of the petal-tetramer as a monomer, Supplementary Figure S1C,D). We estimated six monomers per ring. An important distinction in the methodologies is that sample preparation and staining impact measurements and cannot be compared across methods. These calculations provided independent verification of the PF-8 rings.

To alter the protein dynamics of PF-8, a relatively low concentration of PF-8 was incubated on ice (4 °C). We hypothesize that the population of proteins below <200 nm^2^ represent monomeric/dimeric PF-8 (Figure 2E, below dashed line) and tetramers, hexamers, or dodecamers, which were measured above 200 nm^2^ (dashed line) but below 325 nm^2^ (Figure 2E, dotted line). Over three hours, we detected a measurable difference in median protein areas as well as an upward shift in the population in the dot plot (Figure 2E). The values listed in Table 1 correlate to the dot plots depicted in Figure 2E. Supplementary Table S2 includes the statistical significance of all of the compared groups. Each dot represents the measured area of a single PF-8 protein. Protein concentration had a minimal impact on the median measured PF-8 area. Interestingly, we observed the greatest abundance of smaller proteins (<200 nm^2^) correlated with PF-8 incubated at 4 °C, the shortest duration (0.5 h), or lowest protein concertation (population below dashed line, Figure 2E). Furthermore, the percentage of PF-8 monomers/dimers was greatest at 0.5 h (34.8% of total proteins measured), 100 ng (24.3%), and 4 °C (38.5%, Supplementary Figure S2A,C). All experimental variables examined displayed the hexamers/dodecamers population as the most abundant except PF-8 at 4 °C and the 3 h incubation time (Supplementary Figure S2A,C). As a molecular comparison, the distribution of the areas for GS showed multiple populations, validating our approach in detecting multiple-size protein complexes and/or different geometries (top-down or side) of a non-viral protein. Together, these results suggest time as a critical mediator of PF-8 higher-order oligomer formation (>325 nm^2^).

### 3.2. PF-8 DNA-Binding Affinity to Double-Stranded DNA via DNA Structural Specificity

**Figure 3:**
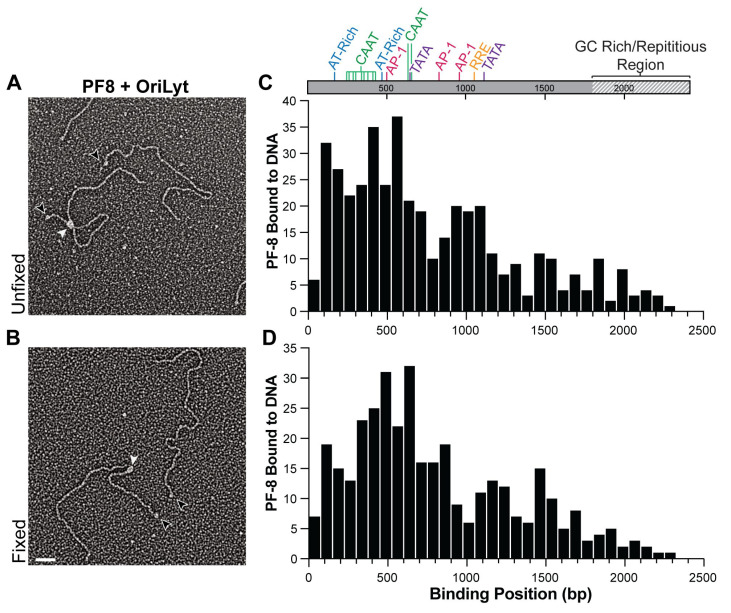
Mapping PF-8 DNA binding locations within KSHV lytic origin DNA. Representative electron micrographs of PF-8 in the (**A**). absence (unfixed) and (**B**). presence (fixed) of glutaraldehyde. White arrowheads denote PF-8 and black arrowheads denote streptavidin. Scale bar = 50 nm (**C**). Positional analysis of PF-8 binding to the OriLyt under unfixed condition (n = 427). (**D**). Positional analysis of PF-8 under binding to OriLyt under fixed conditions (n = 361). Aligned with the histograms is the annotated sequence of the OriLyt. Known sequences that correspond to the x-axis and binding position (bp) are highlighted. Histogram bin sizes are set to 75 bp.

Using our imaging-based approach, we visualized hundreds of individual DNA–protein complexes, which we analyzed to determine PF-8’s DNA binding position and the frequency of molecules bound to specific DNA locations. For our analysis, OriLyt was linearized and labeled with biotin and streptavidin (Figure 3A,B, black arrowhead) and subsequently incubated with PF-8 in the absence (unfixed) and presence (fixed) of glutaraldehyde (Figure 3A,B, white arrowhead). Glutaraldehyde helps stabilize the protein–protein interactions by cross-linking amine groups [44,45]. Fractions from the size exclusion column of the DNA–protein *in vitro* binding reaction were absorbed onto glow-discharged carbon-coated copper mesh grids, shadowed with tungsten, and visualized with TEM (Figure 3A,B). Out of the 427 total positions measured for PF-8 bound to OriLyt under unfixed conditions 53.4% bound between 0 and 675 bp. In the absence of fixative, PF-8 bound to OriLyt between 525 and 600 bp (n = 37) at the highest frequency and regions 375-450 bp (n = 35), which correlates to three CAAT sites and 75–150 bp (n = 32, Figure 3C and Table 2). While in the presence of glutaraldehyde, a comparable number of PF-8 molecules bound between 0 and 675 bp (51.8%, Figure 3D). Out of the 361 total positions measured for PF-8 bound to the OriLyt under fixed conditions, the highest frequency was observed between 600 and 675 bp (n = 32), which corresponds to two CAAT sites and a TATA box. PF-8 also bound to DNA regions 450–525 bp (n = 31), which correlates to a known AP-1 site and an AT-rich region and 375–450 bp (n = 25), which encodes three CAAT sites (Figure 3D and Table 2). PF-8 may preferentially bind to certain CAAT, AP-1 sites, TATA boxes, and AT-rich regions. Another advantage of our approach is the capacity to detect both high and low-frequency binding sites, PF-8 mapped to DNA locations throughout the OriLyt, directly supporting PF-8’s affinity to double-stranded DNA [35]. To determine whether the PF-8 DNA peaks identified in our DNA mapping (Figure 3D) indicate PF-8 sequence specific for known DNA consensus sequences (Supplementary Figure S3) [10,14,15], we introduced several nucleotide mutations (Supplementary Table S1) to OriLyt.

**Figure 4:**
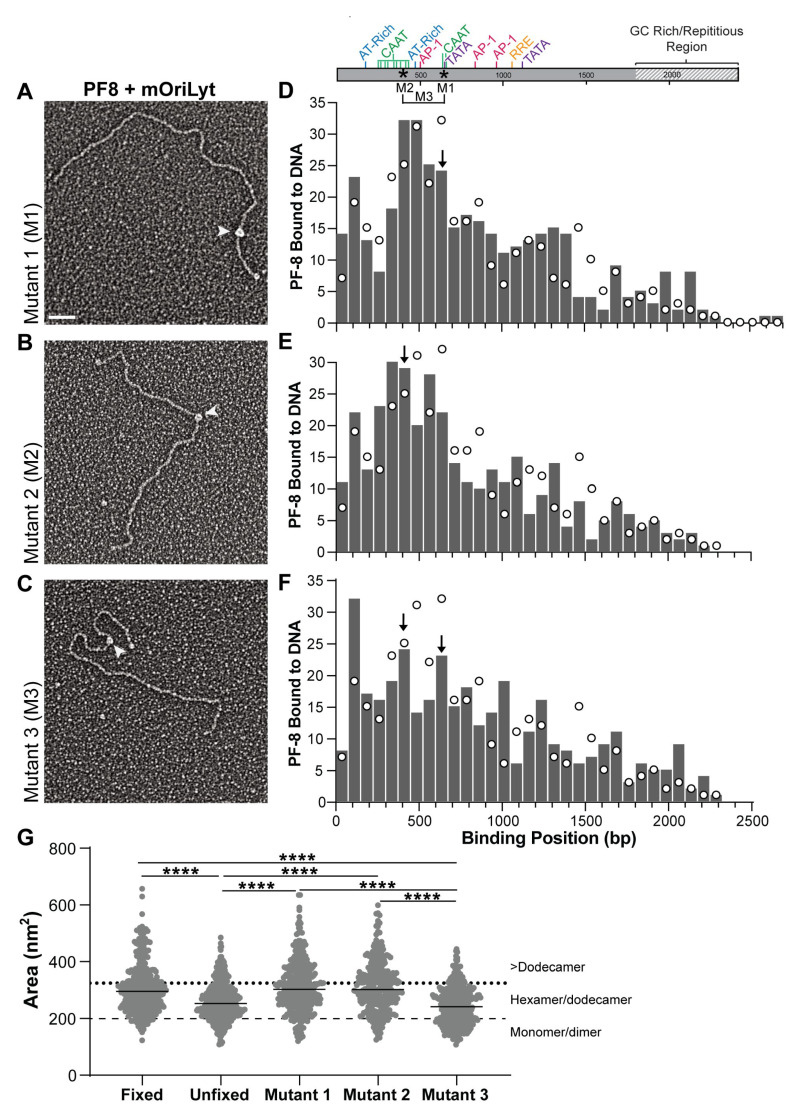
Modulating PF-8 DNA binding locations with nucleotide substitutions within OriLyt. Representative electron micrograph of PF-8 bound to (**A**). mutant 1 (M1), (**B**). mutant 2 (M2), or (**C**). mutant 3 (M3) OriLyt DNA under fixed conditions. Arrowheads denote PF-8. Scale bar = 50 nm. The DNA positional histograms of PF-8 bound to (**D**). mutant 1 (M1), (**E**). mutant 2 (M2), or (**F**). mutant 3 (M3) OriLyt DNA. Arrows denote the peak that contains the mutated nucleotides. Aligned with the histograms is the annotated sequence of the OriLyt with known sequences and locations of mutations. Open circles correlate with the frequency heights for the wild-type OriLyt histogram shown in Figure 3C. (**G**). Dot plot of the areas of PF-8 bound to the different OriLyt DNAs. The median values are shown for each condition by the horizontal line. Dotted and dashed lines represent cutoffs for the different PF-8 oligomers: monomer/dimer, hexamer/dodecamer, and >dodecamer. Kruskal–Wallis with Dunn’s multiple comparison test was used to determine statistical significance for protein area, **** indicates *p*-value < 0.0001.

A subset of DNA-binding proteins will seek and bind to highly conserved DNA consensus sequences (base readout). For example, the transcription factor basic-helix-loop-helix protein Pho4 specifically binds to the E-box sequence within DNA [46,47]. While other DNA-binding proteins have an affinity to specific DNA structural features (shape readout) [47,48]. The HMG box protein is an example that predominately utilizes a shape readout [47]. Shape readout refers to deviations in B-DNA that result in either a local or global 3D shape of the DNA helix. Local shape readout can include narrowing of the minor groove while global shape readout can include DNA bending; however, shape readouts are not limited to only those aspects [49]. To determine which mechanism DNA-binding proteins utilize, mutations can be introduced in the DNA in order to alter local sequences or disrupt the local or global shape [50]. Three mutant OriLyt DNAs were engineered to the preferential binding regions of PF-8. The two CAAT and TATA box motifs contained in the highest frequency peak under fixed conditions were mutated (underlined) from CAAT to CCAT and TATAAA to TAGAAA to generate Mutant 1 (M1). Mutant 2 (M2) was generated by mutating the three CAAT sites to CCAT between 375 and 450 bp, which was the third most frequent peak under fixed conditions. Mutant 3 (M3) had the combined mutations from both M1 and M2. PF-8 was separately incubated with each of the three mutant OriLyt DNAs, visualized with TEM, and analyzed (Figure 4A–C). The frequency of PF-8 was mapped to the DNA binding position (gray bars), and the wild-type OriLyt PF-8 DNA binding frequencies are overlayed as open circles (Figure 3B). The three highest frequency binding peaks PF-8 mapped to M1 were 375–450 bp (n = 32), 450–525 bp (n = 32), and 525–600 bp (n = 25, Figure 4D). The peak that coincided with the mutated CAAT and TATA box motifs had a 2.6% decrease in PF-8 molecules binding to the region compared to the wild-type. The highest frequency peaks PF-8 mapped to M2 were 300–375 bp (n = 30), 375–450 bp (n = 29), and 525–600 bp (n = 28, Figure 4E). The peak that contained the mutated motifs (375–450 bp) had an increase of 1.3% of PF-8 molecules binding to the region compared to the wild-type. The three most frequent peaks mapped for M3 were 75–150 bp (n = 32), 375–450 bp (n = 24), and 600–675 bp (n = 23) (Figure 4F). There was a 2.6% decrease in PF-8 binding observed between 600 and 675 bp compared to the wild-type, which was observed with M1. Between 375 and 450 bp, PF-8 had a 0.4% decrease in binding compared to the wild-type and a 1.7% decrease in binding compared to M2. The two highest peaks mapped for M3 correlated to two out of the three highest peaks for PF-8 under unfixed conditions. Table 2 summarizes the three primary peaks for PF-8 binding to the various DNAs under different conditions. Our mutagenesis approach revealed the prominence of a new peak (75–150 bp) in M3. The mutant OriLyt DNA experiments suggest that DNA nucleotide substitutions may be altering the local or global DNA structure rather than the requirement for a base-specific or consensus sequence driving PF-8 binding. Evidence for a sequence-specific consensus sequence would have greatly diminished the protein binding to the location of mutations. Overall, the mutagenesis strategy successfully altered the PF-8 DNA-binding affinities.

In addition to the DNA positional analysis, we also quantified the protein area when PF-8 was bound to all DNA constructs (Figure 4G). We wanted to determine if association with DNA changed the measured PF-8 area and if fixation or DNA sequence influenced the protein–protein interactions of PF-8. There is a measurable difference in the spread of the IQR between unfixed (215.2–301.7 nm^2^) and fixed (243.2–353.5 nm^2^) with an approximate 50nm^2^ increase in IQR for the fixed group likely because of the absence of a crosslinking reagent reduces the stability of some of the larger protein complexes (Figure 4G and Table 3). PF-8 under unfixed conditions had similar percentages of protein molecules <200 nm^2^, 200–325 nm^2^, and >325 nm^2^ compared to the two-hour time point in the absence of DNA, which is the approximate time it takes for the experimental setup of the DNA–protein setup. There are similar distribution spreads and IQR in the protein area when comparing the fixed group to M1 and M2 suggesting similar PF-8 oligomeric states are interacting with these DNAs. M3 shows an IQR more similar to the unfixed group than the fixed indicating that the DNA mutations present in M3 OriLyt may be influencing the multimeric state of PF-8. Together the single molecular EM analysis revealed subtle changes in PF-8 DNA binding locations and protein areas.

### 3.3. PF-8 Protein Complexes Mediated Changes to OriLyt DNA Architectures

**Figure 5:**
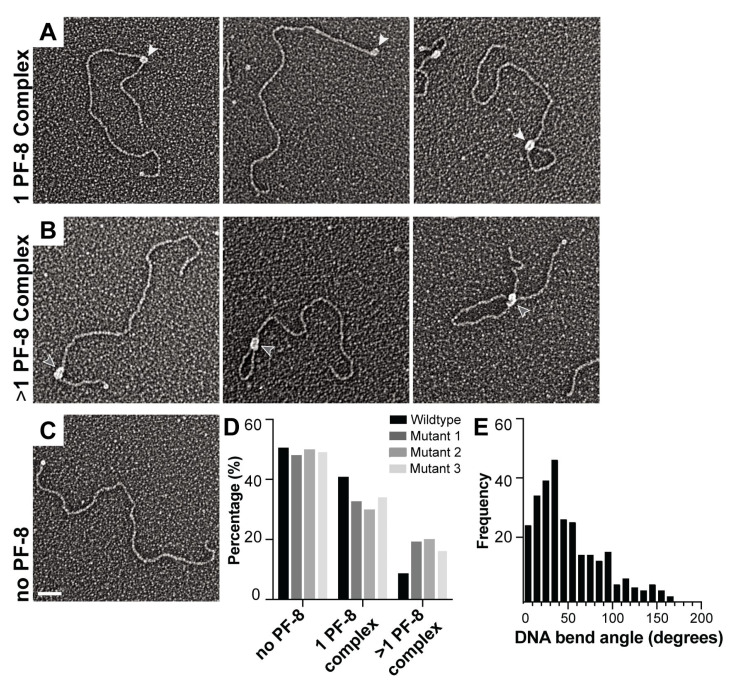
Characterizing OriLyt DNA structures and PF-8 complexes. Representative electron micrographs of OriLyt DNA and PF-8: (**A**). Single PF-8 complexes with linear and looped OriLyt. (**B**). Multiple PF-8 complexes with linear and looped OriLyt. (**C**). OriLyt DNA labeled with streptavidin and no PF-8 associated. White arrowheads denote one PF-8 complex and gray arrowheads denote two PF-8 complexes. Scale bar = 50 nm. (**D**). Quantification of DNA that contains no PF-8 bound, one complex of PF-8 bound, or two complexes of PF-8 bound with the wild-type, mutant 1, mutant 2, and mutant 3 DNAs under fixed conditions. The percentage of DNA molecules was calculated by dividing the number of molecules observed in each category by the total number of DNA molecules quantified and multiplying by 100. **(E**). Histogram of the PF-8 induced DNA bend angles.

With our method, we are able to directly observe different conformations in DNA and protein. For example, we are able to detect if protein binding to DNA is producing multiple loops in the DNA or if protein conformations have uniform or distinct shapes and sizes (area). In order to quantify the OriLyt architecture and protein conformations, we unbiasedly scanned the EM grid and counted the different conformations and architecture present for each type of DNA. We verified that DNA binding did not modify the overall measured DNA length of OriLyt (Supplementary Table S3). This additional analysis provides a comprehensive accounting of the different conformations present in protein-DNA binding reactions. Figure 5A–C shows representative EM images of the different DNA and protein conformations: one complex, multiple complexes, and DNA without PF-8.

The EM micrograph montage includes a variety of examples of PF-8 OriLyt complexes we observed (Figure 5A,B). Representative images include a typical image that we would use to quantify the position and area of PF-8 (Figure 5A left and right panel). We also observed PF-8 binding at one of the ends of the DNA, with some of the end binding resulting in a loop. These PF-8 OriLyt complexes lacking streptavidin end-labeling were omitted from DNA position analysis due to the lack of reference point. PF-8 induced DNA-looping, which indicates that PF-8 is binding to the DNA at two different locations. It is possible that two ring-shaped hexamer complexes that have bound at different locations on the DNA are assembling into a stacked dodecamer (Figure 2D). In addition, we observed two PF-8 complexes that were partially overlapping with one another (Figure 5B). Furthermore, the central pores formed in the petal and ring-shaped structures are both wide enough to accommodate two segments of DNA to pass through (Figure 1C), supporting the PF-8 ring complex structure. We observed a decrease in the one protein complex binding with the mutant DNAs compared to the wild-type and an increase in the presence of the two PF-8 complexes (Figure 5D). The most prominent peak for PF-8-induced bending in the OriLyt was between 30 and 40° (Figure 5E). The eukaryotic processivity factor proliferating cell nuclear antigen (PCNA) encircles the DNA and is known to induce a 40° bend in DNA; thus, PF-8 is perhaps behaving in a similar manner, encircling OriLyt DNA [30].

### 3.4. Multivariable Analysis of PF-8 Protein Area and DNA Binding Position

**Figure 6:**
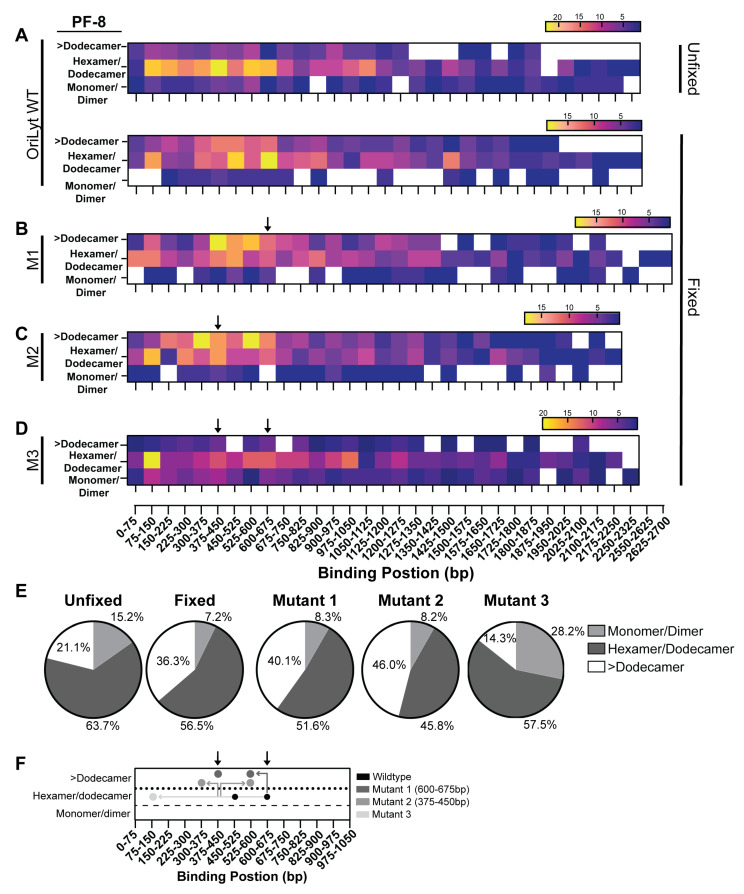
Differential analysis of PF-8 complex area and DNA binding location(s): Heat maps depict the number of PF-8 protein complexes and protein area compared to PF-8 DNA binding locations under (**A**). unfixed or fixed conditions with OriLyt, and fixed conditions with (**B**). mutant 1 (M1), (**C**). mutant 2 (M2), and (**D**). mutant 3 (M3). The protein area measurements were subdivided into three different categories: greater than a dodecamer has an area >325 nm^2^, dodecamer/hexamer has an area between 200 and 325 nm^2^, and a monomer/dimer area <200 nm^2^ left y-axis. The mutations are indicated by arrows above the heat map. (**E**). Pie charts display the proportion of the different oligomeric states of PF-8 under different conditions and with different DNAs. (**F**). Schematic depicting the relationship between PF-8 area (shifts between 3 categories) and DNA binding location (DNA binding region) to wild-type and mutant OriLyt DNA (M1-3). The grey directionality of arrows within the graph indicates changes in binding location and shifts in protein area. Black circles denote hexamer/dodecamer PF-8 highest frequency binding location to wild-type OriLyt, and grey circles represent PF-8 highest frequency binding location to mutant OriLyt (M1-3). The black arrows above the graph indicate mutation locations.

Various DNA-binding proteins can bind to specific sequences when assembled into larger protein complexes [51,52]. For example, the monomer of protelomerase TelK binds to DNA non-specifically while the dimers bind to a target-specific sequence [52]. In addition, we have previously shown that RTA dimers specifically bind to AT-rich and AP-1 sites within the OriLyt [22]. We aimed to determine if certain oligomeric states of PF-8 localize to specific locations on the DNA. In order to answer that question, we constructed heat maps that consist of three variables: binding position, protein area, and frequency. For unfixed conditions, the PF-8 hexamer/dodecamer was the most prevalent at 375–450 bp, 525–600 bp, and 75–150 bp, which correlates with the three most frequent peaks in the histogram (Figure 6A top). The two highest frequency spots for PF-8 under fixed conditions are 600–675 bp and 450–525 bp with an oligomeric state of a hexamer/dodecamer (Figure 6A bottom). The two most frequent locations for mutant 1 were 375–450 bp and 525–600 bp with an oligomeric state greater than a dodecamer (Figure 6B). Mutant 2 revealed that PF-8 preferentially binds to 300–375 bp and 525–600 bp greater than a dodecamer state (Figure 6C). PF-8 binds to mutant 3 with a strong preference at 75–150 bp as a dodecamer/hexamer (Figure 6D). The monomer/dimer population of PF-8 binds non-specifically throughout the length of OriLyt. At least 45% of the PF-8 molecules bound to OriLyt are hexamer/dodecamer (Figure 6E). There is a decrease in the number of molecules greater than dodecamer and an increase in the monomer/dimer population in the unfixed group compared to the fixed. We observed an increase in the greater-than-dodecamer population for mutants 1 and 2. Mutant 3 had the largest percentage of PF-8 molecules in the monomer/dimer range. PF-8 monomer/dimers are less frequently bound to DNA, and their binding appears general throughout the DNA, while ring structures or multiple ring complexes localize to discrete locations. Fixation stabilizes the multiple ring complexes (>dodecamer) bound to DNA. We predict that PF-8 hexameric rings are more likely to bind wild-type OriLyt. This could indicate that monomers initially bind and that the subsequent protein–protein interactions are enhanced by PF-8 binding to DNA (by restricting movement and increasing the probability of protein–protein interactions) in order to promote the ring complexes. Alternatively, ring formation could occur prior to proteins interacting with DNA; however, the PF-8 ring affinity to OriLyt is greater than monomers, which enhances hexamer/dodecamer rings bound to DNA.

Figure 6F highlights the highest frequency box observed in the heatmaps (Figure 6A–D, yellow boxes) for wild-type and mutant OriLyts under fixed conditions. The most abundant area PF-8 bound to two distinct locations on the wild-type OriLyt were in the dodecamer/hexamer range (Figure 6F, black dots). PF-8 bound to mutant 1 OriLyt showed shifted binding preference away from mutant 1 location (600–675 bp) to two new upstream locations and had increased protein areas (Figure 6F, top dark grey dots). PF-8 bound to mutant 2 OriLyt also showed shifted binding preference; however, this occurred both upstream and downstream of the mutant 2 location (375–450 bp). Similar to PF-8 bound to mutant 1, PF-8 bound to mutant 2 also increased protein area into the >dodecamer range (Figure 6F, grey dots). Finally, PF-8 bound to mutant 3 OriLyt, which possesses both mutant 1 and mutant 2 mutations, revealed that PF-8 dramatically shifted upstream closer to the biotin/streptavidin labeled end of the DNA. The observed protein area and DNA location shift we captured when comparing the four different OriLyt fragments revealed the dynamic DNA binding activity of PF-8. These data also support PF-8 area and DNA binding is influenced by DNA sequence and/or shape.

## 4. Discussion

We utilized a single-molecule approach with TEM to characterize PF-8 protein–protein and DNA–protein interactions. The advantage of this technique over more traditional gel-based assays is that heterogenous populations can be observed and individual populations can be subdivided and further analyzed. While more traditional gel-based assays provide a bulk analysis and reveal the most prominent species. Additionally, a direct imaging approach provides both quantitative and qualitative data which when compiled together allows for a comprehensive characterization. We investigated the oligomeric state of PF-8 and PF-8 bound to KSHV lytic origin DNA. In addition, we mapped the binding frequency of PF-8 to the wild-type KSHV OriLyt and three mutant OriLyts. Furthermore, we quantified the different DNA architectures and protein phenotypes among the wild-type OriLyt and three mutant OriLyts.

Most studies suggest that PF-8 forms a dimer in solution and when bound to DNA, with a few studies suggesting a tetramer state [19,53]. No studies have investigated the oligomeric state of full-length PF-8 at the single-molecule level. The crystal structure of a truncated PF-8 lacking the C-terminus revealed that it can form a dimer when unbound to DNA. EBV’s processivity factor BMRF1 is known to form dimers and tetramers based on a truncated crystal structure [31,32]. In addition, ring-shaped structures with a central pore were observed when BMRF1 was stained with uranyl acetate and were hypothesized to be a hexamer. The central pore and complex measurements (pore: 5.3 ± 0.8 nm complex: 15.5 ± 0.8 nm) were similar to PF-8 (Table 4) [33]. Based on the denaturing gel, semi-native gel, and tungsten rotary shadowing data, PF-8 may form a similar oligomeric state as GS. GS is known to form a dodecamer through the stacking of two hexamers on top of each other with a central pore in the center [54]. The top-down view is likely the most prominent conformation, which is similar to GS, suggesting more of a hexamer formation as opposed to the addition of a side view, which suggests a dodecamer state through the stacking of two hexamer rings. Since the central pore is large enough to accommodate the double helix of DNA, we hypothesize that the PF-8 is encircling the DNA. Some processivity factors such as PCNA are known to have a central pore that DNA can pass through [30]. The formation of a hexamer and/or dodecamer state could be a unique characteristic among gamma herpesviruses processivity factors; specifically, a ring conformation that encircles DNA, leading to DNA bending at a certain angle. Several processivity factors that encircle DNA are known to cause DNA bending angles similar to our observed results, adding further support to our hypothesis [30].

Although PF-8 is required for the efficient replication of the KSHV genome, it is not the only protein capable of binding to OriLyt DNA. RTA and DNA polymerase are both lytic KSHV replication proteins that are known to interact with PF-8 and bind to DNA [13,26]. Rossetto et al. showed that PF-8 binding to the CAAT palindromes was enhanced with the presence of RTA using a ChIP assay with PCR primers flanking the CAAT palindrome region [26]. The addition of other proteins that interact with PF-8 may alter the sequence-specific region that PF-8 binds. In addition, our method utilizing TEM allows for several populations to be studied, while ChIP coupled with PCR can only indicate whether a protein is binding to a particular region. It does not provide information on the frequency or if the protein is binding to other regions at a higher frequency. Furthermore, PF-8 could bind to those regions and wait for the other proteins to associate with the DNA so that DNA replication can be initiated. C/EBP proteins are transcription factors that are known to bind to CAAT sequences [55]. C/EBP proteins have been previously shown to associate with other proteins that are known to be involved in lytic DNA replication, such as K8 and RTA [56,57]. PF-8 could be dwelling in this area while other proteins are recruited to the area to form the pre-initiation DNA replication complex that requires RTA and K8 [58].

During protein–DNA binding, PF-8 scans the OriLyt sequence for distinct DNA shape readouts. PF-8 rings are more likely bound to 1-675 bp region and eventually settle into specific regions. Introducing DNA mutations modified PF-8 binding locations as well as PF-8-measured areas (Figure 6F). The presence of mutant 1 nucleotide substitutions enhanced the accumulation of PF-8 complexes with greater areas to localize more upstream proximal to the wild-type OriLyt hexameric rings. PF-8 bound to mutant 2 shifted the PF-8 into the higher area category (above the dotted line) and shifted binding upstream and downstream of the mutation site. The accumulation of all six mutations (mutant 1 plus mutant 2) resulted in an upstream shift of hexameric PF-8 rings (between dashed and dotted lines) to localize to the 75–150 bp region. Future studies will aim to decipher the shape readouts, specifically the contributions of GC- and AT-composition, major and minor groove frequency, and span additional structural features known to impact protein–DNA-binding affinities [49].

**Figure 7:**
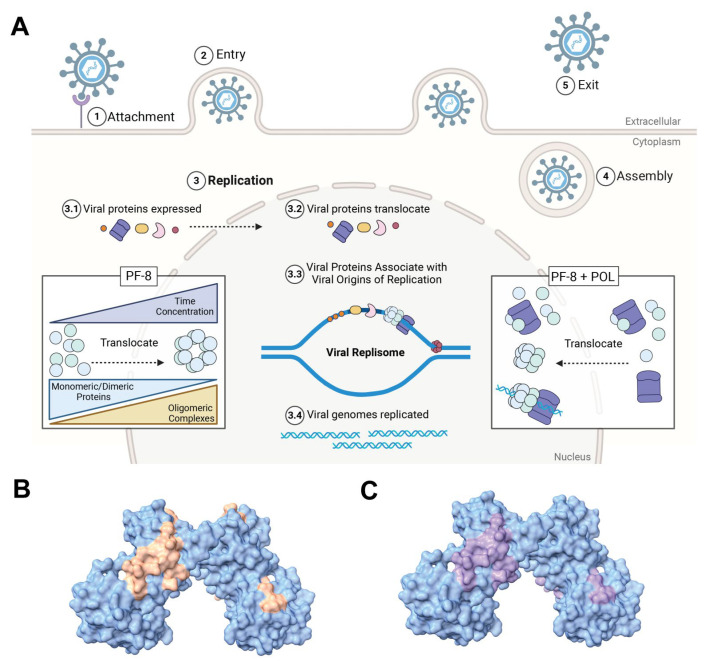
Models of PF-8’s structures, function, and localization during KSHV lytic replication. (**A**). During KSHV lytic DNA replication, PF-8 is translated into the cytoplasm and subsequently translocated into the nucleus. The effective concentration of PF-8 in the nucleus increases the propensity of PF-8 to form ring complexes. PF-8 is required for KSHV DNA polymerase (POL) translocation into the nucleus. Once KSHV DNA replication proteins are localized within the nucleus, the viral replisome assembles to initiate viral replication. Light green and blue circles indicate PF-8 and purple protein denotes POL. Crystal structure [3HSL] of PF-8 dimers depicting (**B**). identified dimerization domains (orange) and (**C**). DNA polymerase binding domain (purple).

Taken together, our findings add key insights into explaining PF-8 activity in the context of the infectious cycle. During the lytic phase, PF-8 is first translated in the cytoplasm, forms mostly monomers and dimers, and, subsequently, localizes to the nucleus, which is the site of viral DNA replication (Figure 7A). As the lytic cycle progresses, the concentration of PF-8 in the cytoplasm decreases and increases in the nucleus. Furthermore, the volume of the nucleus is much less compared to the cytoplasm, further increasing the effective concentration of PF-8 in the nucleus. The effective concentration of PF-8 may need to be at a high level to promote the higher oligomeric states to be achieved (Figure 2E). In addition, those higher oligomeric states may be needed for PF-8 to preferentially bind to specific regions within the OriLyt for processivity function and efficient lytic DNA replication. The dimerization domains (orange) and KSHV DNA polymerase (purple) of PF-8 are pseudo colored on space filling model of an *E. coli* purified PF-8 (PDB:3HSL, Figure 7B,C) overlap. PF-8 monomers/dimers in the cytoplasm would bind KSHV POL and translocate the POL into the nucleus. KSHV POL lacks its own nuclear localization sequence and requires PF-8 binding to enter the nucleus [59]. PF-8 dimers would contain two DNA POL binding sites and enhance POL nuclear localization. Within the nucleus, we believe three dimers oligomerize to form hexameric rings. The location of the DNA binding domains supports our model where the viral DNA is threaded through the central pore and a greater number of PF-8 proteins in a ring confirmation would encode more DNA-binding sites compared to monomers or dimers.

Our approach to characterizing PF-8 at the single molecule utilizes an *in vitro* system with purified components. A limitation of using an *in vitro* approach is that the system lacks cellular or other viral components that may influence DNA binding or protein–protein interactions. Studying the individual components of lytic DNA replication can help provide a basis or initial understanding of how these components behave by themselves and may help guide *in vivo* or cellular studies. Future studies include performing a combinational analysis of PF-8 with other lytic DNA replication proteins that interact with PF-8 such as RTA or DNA polymerase.

## Figures and Tables

**Table 1:** KSHV processivity factor and glutamine synthase protein area.

Proteins	Median	IQR (Lower-Upper)	n = Proteins
**PF-8**			
4 °C	314.6 nm^2^	215.2–301.7 nm^2^	122
0.5 h	243.3 nm^2^	145.7–279.1 nm^2^	335
1 h	280.2 nm^2^	244.7–305.9 nm^2^	293
2 h	295.9 nm^2^	258–326.4 nm^2^	311
3 h	347.8 nm^2^	266.9–409.8 nm^2^	314
100 ng	274.8 nm^2^	203.1–308.6 nm^2^	317
200 ng	271.2 nm^2^	236.5–298.6 nm^2^	325
400 ng	277.5 nm^2^	241.5–310.9 nm^2^	351
800 ng	263.6 nm^2^	232.9–298.5 nm^2^	430
*Molecular protein standard*			
**GS**	306.8 nm^2^	250.1–338.0 nm^2^	1112

ng = nanogram, nm = nanometer.

**Table 2:** Preferential binding locations of KSHV processivity factor.

	Primary Peak 1		Peak 2		Peak 3	
*DNA*	*Position*	*Motifs*	*Position*	*Motifs*	*Position*	*Motifs*
OriLyt unfixed	525–600 bp (n = 37)		375–450 bp (n = 35)	3 CAAT	75–150 bp (n = 32)	
OriLyt fixed	600–675 bp (n = 32)	2 CAAT, TATA box	450–525bp (n = 31)	AT-rich, AP-1	375–450 bp (n = 25)	3 CAAT
Mutant 1 fixed	375–450 bp (n = 32)	3 CAAT	450–525 bp (n = 32)	AT-rich, AP-1	525–600 bp (n = 25)	
Mutant 2 fixed	300–375 bp (n = 30)	3 CAAT	375–450 bp (n = 29)	3 CAAT	525–600 bp (n = 28)	
Mutant 3 fixed	75–150 bp (n = 32)		375–450 bp (n = 24)	3 CAAT	600–675 bp (n = 23)	2 CAAT, TATA box

**Table 3:** KSHV Processivity Factor Protein Area Bound to DNA.

DNA/Condition	Median	IQR (Lower-Upper)	n = Proteins
OriLyt unfixed	253.0 nm^2^	215.2–301.7 nm^2^	370
OriLyt fixed	295.4 nm^2^	243.2–353.5 nm^2^	343
Mutant 1 OriLyt	302.9 nm^2^	251.7–369.6 nm^2^	384
Mutant 2 OriLyt	302.5 nm^2^	250.6–362.1 nm^2^	311
Mutant 3 OriLyt	241.6 nm^2^	190.6–296.2 nm^2^	298

**Table 4:** Measured and Calculated Dimensions PF-8 Pore and PF-8 Complex.

Protein	Predicted Oligomeric State	Method	Pore Diameter (nm ± SD)	Complex Diameter (nm ± SD)	Reference
BMRF1	Hexamer	Negative staining	5.3 ± 0.8	15.5 ± 0.8	AM Makhov et al. [33]
Truncated BMRF1	Tetramer	Crystal structure (PDB:2Z0L)	4.9	11.2	K Murayama et al. [31] S Nakayama et al. [32]
GS	Dodecamer	Negative staining	N/A	14.0	G Harth et al. [38]
GS	Dodecamer	Crystal structure (PDB: 8PVG)	4.0-5.0	14.0	PC Huang et al. [54]
PF-8	Tetramer	Negative staining	5.0 ± 0.47	14.5 ± 0.95	-
PF-8	Hexamer	Negative staining	6.1 ± 0.89	13.3 ± 0.96	-

## Data Availability

The raw data supporting the conclusions of this article will be made available by the authors upon request.

## Supplementary Materials

The following supporting information can be downloaded at: https://www.mdpi.com/article/10.3390/v17020190/s1, Figure S1: Area measurements and diameter calculations for PF-8 and GS; Figure S2: Proportion of the different multimeric states of PF-8 in the absence of DNA; Figure S3: Annotated OriLyt sequence; Table S1: OriLyt site-directed mutagenesis primers; Table S2: Statistical significance of protein areas in the absence of DNA; Table S3: OriLyt DNA full-length measurements.

## Abbreviations

The following abbreviations are used in this manuscript:

KSHV	Kaposi’s Sarcoma Herpesvirus
PF-8	Processivity Factor-8
POL	DNA polymerase
OriLyt	Origin of replication
EBV	Epstein–Barr Virus
KS	Kaposi Sarcoma
ORF	Open reading frame
RRE	RTA response element
TEM	Transmission electron microscopy
GS	Glutamine synthetase
M1-3	Mutant 1, Mutant 2, or Mutant 3
nm	nanometers
ng	nanograms

## References

[1] Cohan B., Frappier L. (2021). Herpesvirus DNA Polymerase Processivity Factors: Not Just for DNA Synthesis. Virus Res..

[2] Van Lint A.L., Knipe D.M. (2019). Herpesviruses. Encycl. Microbiol..

[3] Li S., Bai L., Dong J., Sun R., Lan K., Cai Q., Yuan Z., Lan K. (2017). Kaposi’s Sarcoma-Associated Herpesvirus: Epidemiology and Molecular Biology. Infectious Agents Associated Cancers: Epidemiology and Molecular Biology Advances in Experimental Medicine and Biology.

[4] Naimo E., Zischke J., Schulz T.F. (2021). Recent Advances in Developing Treatments of Kaposi’s Sarcoma Herpesvirus-Related Diseases. Viruses.

[5] Polizzotto M.N., Uldrick T.S., Hu D., Yarchoan R. (2012). Clinical Manifestations of Kaposi Sarcoma Herpesvirus Lytic Activation: Multicentric Castleman Disease (KSHV-MCD) and the KSHV Inflammatory Cytokine Syndrome. Front. Microbiol..

[6] Myoung J., Ganem D. (2011). Generation of a Doxycycline-Inducible KSHV Producer Cell Line of Endothelial Origin: Maintenance of Tight Latency with Efficient Reactivation upon Induction. J. Virol. Methods.

[7] Minhas V., Wood C. (2014). Epidemiology and Transmission of Kaposi’s Sarcoma-Associated Herpesvirus. Viruses.

[8] Schulz T.F. (2021). Kaposi’s Sarcoma-Associated Herpesvirus-Antiviral Treatment. New Drug Development for Known and Emerging Viruses.

[9] Coen N., Duraffour S., Snoeck R., Andrei G. (2014). KSHV Targeted Therapy: An Update on Inhibitors of Viral Lytic Replication. Viruses.

[10] Wang Y., Li H., Chan M.Y., Zhu F.X., Lukac D.M., Yuan Y. (2004). Kaposi’s Sarcoma-Associated Herpesvirus Ori—Lyt -Dependent DNA Replication: Cis -Acting Requirements for Replication and Ori—Lyt -Associated RNA Transcription. J. Virol..

[11] Cai Q., Verma S.C., Lu J., Robertson E.S. (2010). Molecular Biology of Kaposi’s Sarcoma-Associated Herpesvirus and Related Oncogenesis. Adv. Virus Res..

[12] Zhou X., Liao Q., Ricciardi R.P., Peng C., Chen X. (2010). Kaposi’s Sarcoma-Associated Herpesvirus Processivity Factor-8 Dimerizes in Cytoplasm before Being Translocated to Nucleus. Biochem. Biophys. Res. Commun..

[13] Chen L.-W., Wang S.-S., Chen L.-Y., Huang H.-Y., He S., Hung C.-H., Lin C.-L., Chang P.-J. (2023). Interaction and Assembly of the DNA Replication Core Proteins of Kaposi’s Sarcoma-Associated Herpesvirus. Microbiol. Spectr..

[14] Aucoin D.P., Colletti K.S., Xu Y., Cei S.A., Pari G.S. (2002). Kaposi’s Sarcoma-Associated Herpesvirus (Human Herpesvirus 8) Contains Two Functional Lytic Origins of DNA Replication. J. Virol..

[15] Lin C.L., Li H., Wang Y., Zhu F.X., Kudchodkar S., Yuan Y. (2003). Kaposi’s Sarcoma-Associated Herpesvirus Lytic Origin (Ori-Lyt)-Dependent DNA Replication: Identification of the Ori-Lyt and Association of K8 BZip Protein with the Origin. J. Virol..

[16] Aneja K.K., Yuan Y. (2017). Reactivation and Lytic Replication of Kaposi’s Sarcoma-Associated Herpesvirus: An Update. Front. Microbiol..

[17] Lin K., Dai C.Y., Ricciardi R.P. (1998). Cloning and Functional Analysis of Kaposi’s Sarcoma-Associated Herpesvirus DNA Polymerase and Its Processivity Factor. J. Virol..

[18] Purushothaman P., Dabral P., Gupta N., Sarkar R., Verma S.C. (2016). KSHV Genome Replication and Maintenance. Front. Microbiol..

[19] Gutierrez I.V., Sarkar P., Rossetto C.C. (2021). Kaposi’s Sarcoma-Associated Herpesvirus Processivity Factor, ORF59, Binds to Canonical and Linker Histones, and Its Carboxy Terminus Is Dispensable for Viral DNA Synthesis. J. Virol..

[20] Travis J.K., Costantini L.M. (2024). Inhibiting KSHV Replication by Targeting the Essential Activities of KSHV Processivity Protein, PF-8. J. Med. Virol..

[21] Baltz J.L., Filman D.J., Ciustea M., Silverman J.E.Y., Lautenschlager C.L., Coen D.M., Ricciardi R.P., Hogle J.M. (2009). The Crystal Structure of PF-8, the DNA Polymerase Accessory Subunit from Kaposi’s Sarcoma-Associated Herpesvirus. J. Virol..

[22] Calhoun J.C., Damania B., Griffith J.D., Costantini L.M. (2023). Electron Microscopy Mapping of the DNA-Binding Sites of Monomeric, Dimeric, and Multimeric KSHV RTA Protein. J. Virol..

[23] Arthur C.P., Ciferri C. (2019). High-Throughput Protein Analysis Using Negative Stain Electron Microscopy and 2D Classification. Methods in Molecular Biology.

[24] Tsutakawa S.E., Sarker A.H., Ng C., Arvai A.S., Shin D.S., Shih B., Jiang S., Thwin A.C., Tsai M.-S., Willcox A. (2020). Human XPG Nuclease Structure, Assembly, and Activities with Insights for Neurodegeneration and Cancer from Pathogenic Mutations. Proc. Natl. Acad. Sci. USA.

[25] McDowell M.E., Purushothaman P., Rossetto C.C., Pari G.S., Verma S.C. (2013). Phosphorylation of Kaposi’s Sarcoma-Associated Herpesvirus Processivity Factor ORF59 by a Viral Kinase Modulates Its Ability To Associate with RTA and Ori Lyt. J. Virol..

[26] Rossetto C.C., Susilarini N.K., Pari G.S. (2011). Interaction of Kaposi’s Sarcoma-Associated Herpesvirus ORF59 with OriLyt Is Dependent on Binding with K-Rta. J. Virol..

[27] DNA Packaging: Nucleosomes and Chromatin|Learn Science at Scitable. https://www.nature.com/scitable/topicpage/dna-packaging-nucleosomes-and-chromatin-310/.

[28] Beckwitt E.C., Kong M., Van Houten B. (2018). Studying Protein-DNA Interactions Using Atomic Force Microscopy. Semin. Cell Dev. Biol..

[29] Pettersen E.F., Goddard T.D., Huang C.C., Meng E.C., Couch G.S., Croll T.I., Morris J.H., Ferrin T.E. (2021). UCSF ChimeraX: Structure Visualization for Researchers, Educators, and Developers. Protein Sci..

[30] Dieckman L.M., Freudenthal B.D., Todd Washington M. (2012). PCNA Structure and Function: Insights from Structures of PCNA Complexes and Post-Translationally Modified PCNA. Subcell. Biochem..

[31] Murayama K., Nakayama S., Kato-Murayama M., Akasaka R., Ohbayashi N., Kamewari-Hayami Y., Terada T., Shirouzu M., Tsurumi T., Yokoyama S. (2009). Crystal Structure of Epstein-Barr Virus DNA Polymerase Processivity Factor BMRF1. J. Biol. Chem..

[32] Nakayama S., Murata T., Yasui Y., Murayama K., Isomura H., Kanda T., Tsurumi T. (2010). Tetrameric Ring Formation of Epstein-Barr Virus Polymerase Processivity Factor Is Crucial for Viral Replication. J. Virol..

[33] Makhov A.M., Subramanian D., Holley-Guthrie E., Kenney S.C., Griffith J.D. (2004). The Epstein-Barr Virus Polymerase Accessory Factor BMRF1 Adopts a Ring-Shaped Structure as Visualized by Electron Microscopy. J. Biol. Chem..

[34] Serec K., Šegedin N., Krajačić M., Babić S.D. (2021). Conformational Transitions of Double-Stranded Dna in Thin Films. Appl. Sci..

[35] Ruby Chan S., Chandran B. (2000). Characterization of Human Herpesvirus 8 ORF59 Protein (PF-8) and Mapping of the Processivity and Viral DNA Polymerase-Interacting Domains. J. Virol..

[36] Zhang Q., Holley-Guthrie E., Dorsky D., Kenney S. (1999). Identification of Transactivator and Nuclear Localization Domains in the Epstein-Barr Virus DNA Polymerase Accessory Protein, BMRF1. J. Gen. Virol..

[37] Colombo G., Villafranca J.J. (1986). Amino Acid Sequence of Escherichia Coli Glutamine Synthetase Deduced from the DNA Nucleotide Sequence. J. Biol. Chem..

[38] Harth G., Clemens D.L., Horwitz M.A. (1994). Glutamine Synthetase of Mycobacterium Tuberculosis: Extracellular Release and Characterization of Its Enzymatic Activity (Tuberculosis/Nitrogen Metabolism/Patbogenesis/Ammonla Regulation). Proc. Natl. Acad. Sci. USA.

[39] Robinson L.Z., Reixach N. (2014). Quantification of Quaternary Structure Stability in Aggregation-Prone Proteins under Physiological Conditions: The Transthyretin Case. Biochemistry.

[40] Travis B.A., Peck J.V., Salinas R., Dopkins B., Lent N., Nguyen V.D., Borgnia M.J., Brennan R.G., Schumacher M.A. (2022). Molecular Dissection of the Glutamine Synthetase-GlnR Nitrogen Regulatory Circuitry in Gram-Positive Bacteria. Nat. Commun..

[41] Niepmann M., Zheng J. (2006). Discontinuous Native Protein Gel Electrophoresis. Electrophoresis.

[42] Nowakowski A.B., Wobig W.J., Petering D.H. (2014). Native SDS-PAGE: High Resolution Electrophoretic Separation of Proteins with Retention of Native Properties Including Bound Metal Ions. Metallomics.

[43] McMullan G., Naydenova K., Mihaylov D., Yamashita K., Peet M.J., Wilson H., Dickerson J.L., Chen S., Cannone G., Lee Y. (2023). Structure Determination by CryoEM at 100 KeV. Proc. Natl. Acad. Sci. USA.

[44] Müller L., Salman S., Hoppe T. (2024). Chemical Cross-Linking to Study Protein Self-Assembly in Cellulo. STAR Protoc..

[45] Migneault I., Dartiguenave C., Bertrand M.J., Waldron K.C. (2004). Glutaraldehyde: Behavior in Aqueous Solution, Reaction with Proteins, and Application to Enzyme Crosslinking. Biotechniques.

[46] McDonald R.J., Kahn J.D., Maher L.J. (2006). DNA Bending by BHLH Charge Variants. Nucleic Acids Res..

[47] Slattery M., Zhou T., Yang L., Dantas Machado A.C., Gordân R., Rohs R. (2014). Absence of a Simple Code: How Transcription Factors Read the Genome. Trends Biochem. Sci..

[48] Lin M., Guo J.T. (2019). New Insights into Protein-DNA Binding Specificity from Hydrogen Bond Based Comparative Study. Nucleic Acids Res..

[49] Rohs R., Jin X., West S.M., Joshi R., Honig B., Mann R.S. (2010). Origins of Specificity in Protein-DNA Recognition. Annu. Rev. Biochem..

[50] Chiu T.P., Xin B., Markarian N., Wang Y., Rohs R. (2020). TFBSshape: An Expanded Motif Database for DNA Shape Features of Transcription Factor Binding Sites. Nucleic Acids Res..

[51] Siggers T., Gordân R. (2014). Protein-DNA Binding: Complexities and Multi-Protein Codes. Nucleic Acids Res..

[52] Landry M.P., Zou X., Wang L., Huang W.M., Schulten K., Chemla Y.R. (2013). DNA Target Sequence Identification Mechanism for Dimer-Active Protein Complexes. Nucleic Acids Res..

[53] Chen X., Lin K., Ricciardi R.P. (2004). Human Kaposi’s Sarcoma Herpesvirus Processivity Factor-8 Functions as a Dimer DNA Synthesis. J. Biol. Chem..

[54] Huang P.C., Chen S.K., Chiang W.H., Ho M.R., Wu K.P. (2022). Structural Basis for the Helical Filament Formation of Escherichia Coli Glutamine Synthetase. Protein Sci..

[55] Bakker O., Parker M.G. (1991). CAAT/Enhancer Binding Protein Is Able to Bind to ATF/CRE Elements. Nucleic Acids Res..

[56] Wang S.E., Wu F.Y., Fujimuro M., Zong J., Hayward S.D., Hayward G.S. (2003). Role of CCAAT/Enhancer-Binding Protein Alpha (C/EBPα) in Activation of the Kaposi’s Sarcoma-Associated Herpesvirus (KSHV) Lytic-Cycle Replication-Associated Protein (RAP) Promoter in Cooperation with the KSHV Replication and Transcription Activator (RTA) and RAP. J. Virol..

[57] Wang S.E., Wu F.Y., Chen H., Shamay M., Zheng Q., Hayward G.S. (2004). Early Activation of the Kaposi’s Sarcoma-Associated Herpesvirus RTA, RAP, and MTA Promoters by the Tetradecanoyl Phorbol Acetate-Induced AP1 Pathway. J. Virol..

[58] Lukac D.M., Yuan Y. (2007). Reactivation and Lytic Replication of KSHV. Human Herpesviruses: Biology, Therapy, and Immunoprophylaxis.

[59] Chen Y., Ciustea M., Ricciardi R.P. (2005). Processivity Factor of KSHV Contains a Nuclear Localization Signal and Binding Domains for Transporting Viral DNA Polymerase into the Nucleus. Virology.

