# Supplementary material for: DNA-Binding Activities of KSHV DNA Polymerase Processivity Factor (PF-8) Complexes
Source: Viruses. 2025 Jan 29;17(2):190. doi: 10.3390/v17020190 (PMC11860742; doi:10.3390/v17020190)
Supplement: Supplementary file 1 [file viruses-17-00190-s001.zip › viruses-3406044-supplementary.pdf]

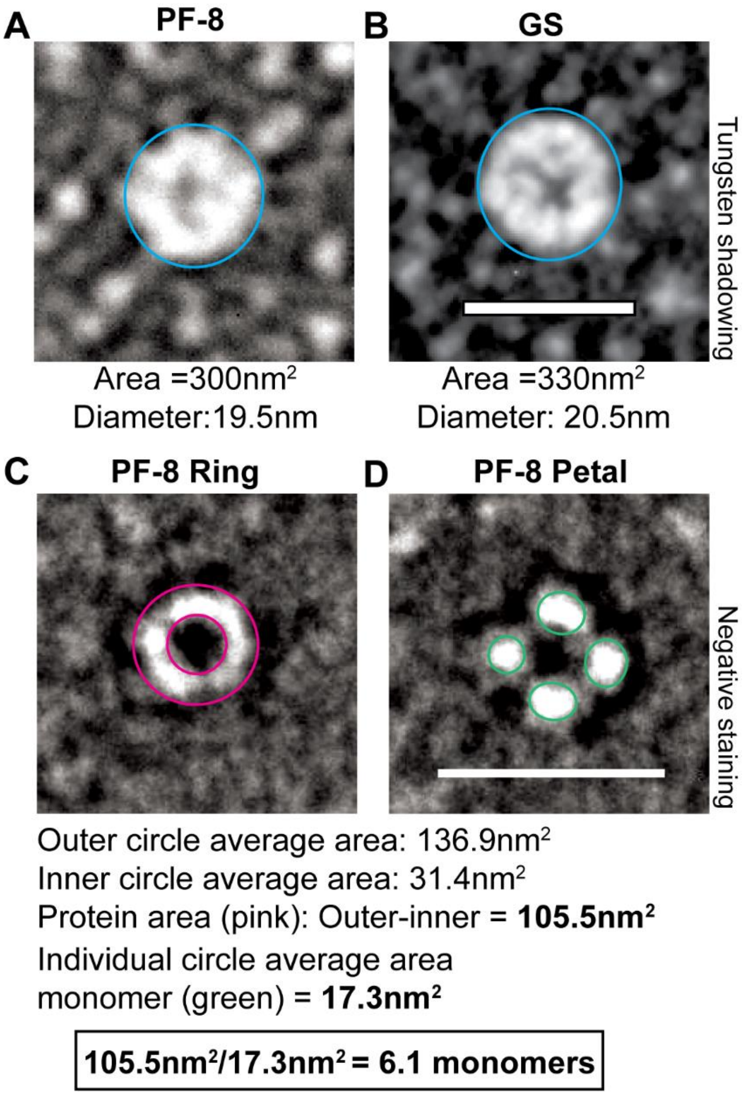

Supplemental Figure S1: Area measurements and diameter calculations for PF-8 and GS. Representative electron micrograph of **A.** PF-8 and **B.** GS prepared with tungsten shadowing. Blue circle represents area measured for each protein. Diameters were calculated assuming the area of a circle. Micrographs of PF-8 **C.** ring and **D.** petal structures prepared with negative staining. Interior and exterior circles denoted in magenta. Individual PF-8 area outlined in green. Measured areas were used to approximate 6 PF-8 monomers per a PF-8 ring. Scale bars=25nm.

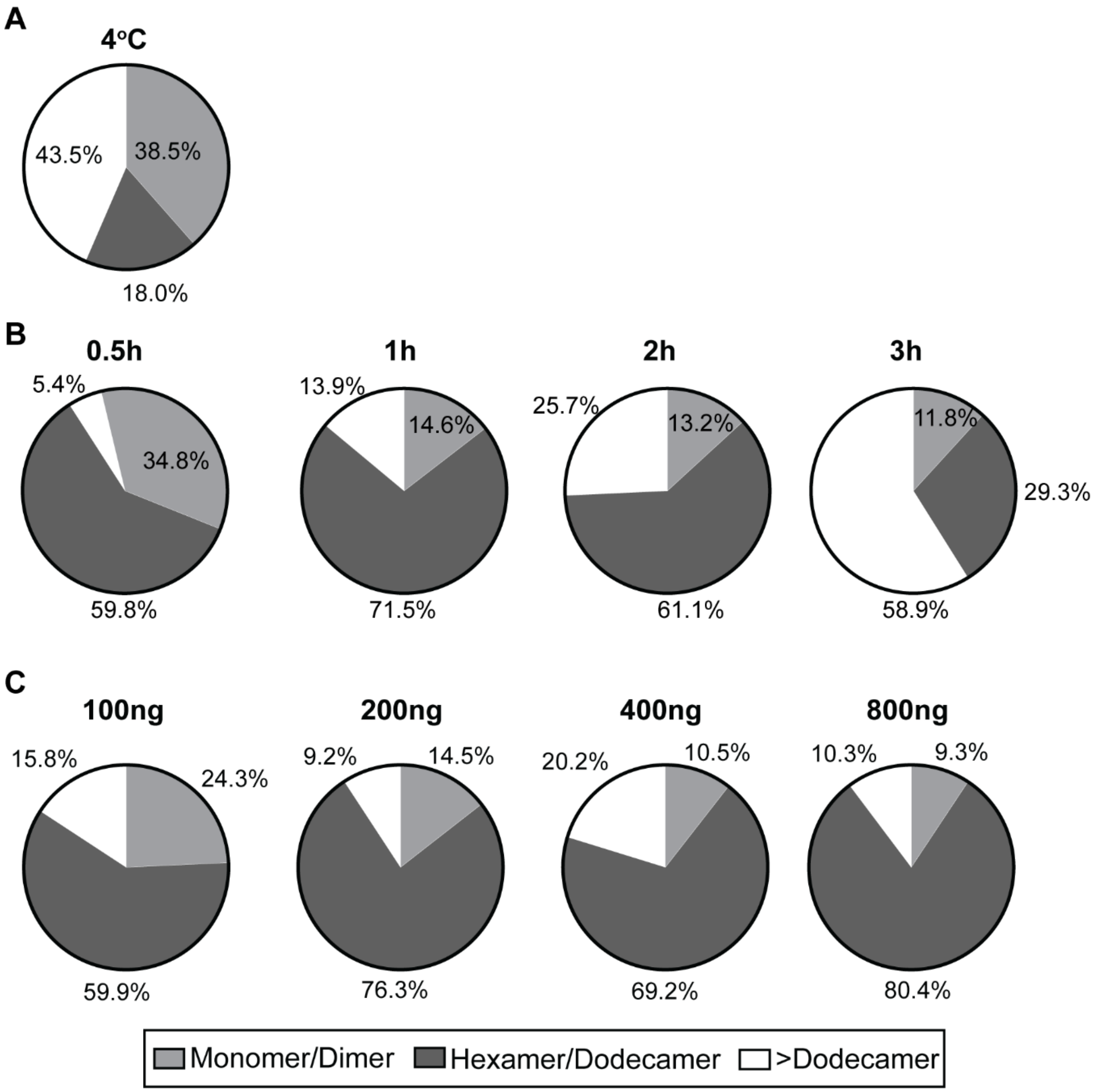

Supplemental Figure S2: Proportion of the different multimeric states of PF-8 in the absence of DNA. A. PF-8 incubated at 4°C. **B.** PF-8 at a range of room temperature incubation times (0.5-3 hours). **C**. PF-8 at a range of amounts (100-800ng) incubated at room temperature. The multimeric states were subdivided by area measurements: >200nm^2 (monomer/dimer), 200-325nm^2 (hexamer/dodecamer), and >325nm^2 (>dodecamer).

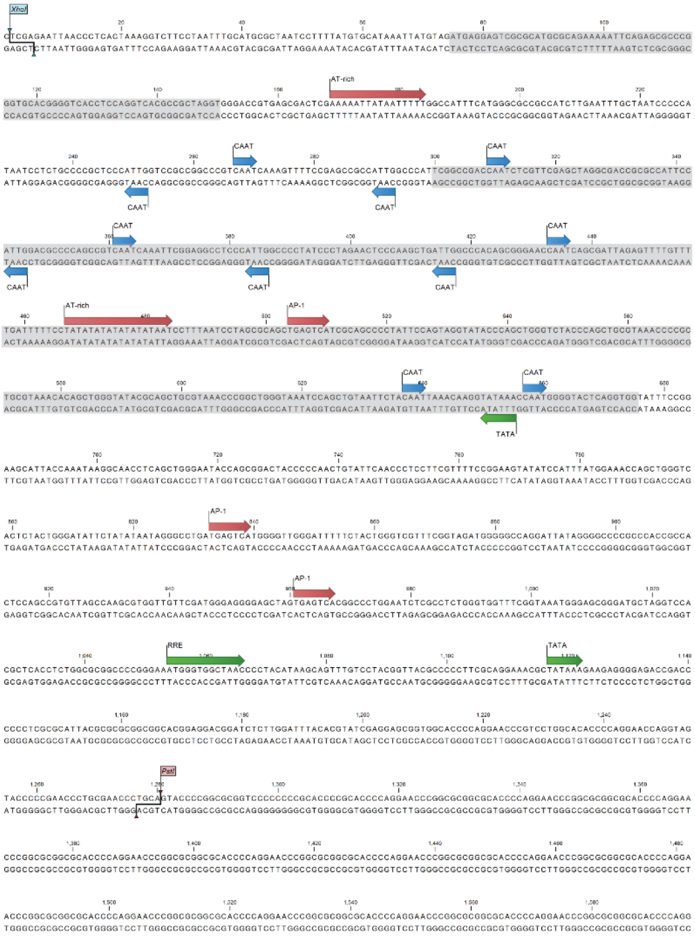

XhoI
AT-rich
CAAT
AP-1
TATA
RRE
PstI

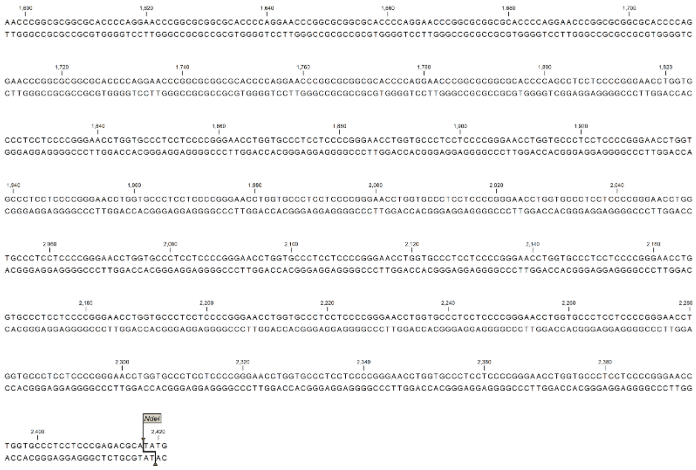

NdeI

Supplemental Figure S3: Annotated OriLyt sequence. The nucleotide sequence of the OriLyt is displayed with the the AT-rich, CAAT, AP-1, TATA box, RRE, and restriction sites annotated. Biotin 14-CTP was incorporated into the OriLyt sequence at position four. The gray box denotes the preferential binding region of PF-8 to the wildtype or mutants 1-3.

Supplementary Table S1. OriLyt Site Directed Mutagenesis Primers

**Mutants**	**Motif**	**Motif Sequence**	**Forward Primer**	**Reverse Primer**
M1	CAAT	652-655	5’-CCTGAGTACCCCAT<br>GGGTTTATACCTTG-3’	5’-CAAGGTATAAACCCAT<br>GGGGTACTCAGG-3’
	TATA box	645-650	5’-CCCCATGGGTTTCT<br>ACCTTGTTTAATGG-3’	5’-CCATTAAACAAGGTAG<br>AAACCCATGGGG-3’
	CAAT	632-635	5’-TACCTTGTTTAATGG<br>TAGAATTACAGCTGG-3’	5-CCAGCTGTAATTCTAC<br>CATTAAACAAGGTA-3’
M2	CAAT	428-431	5’-CTAATCGCTGATGG<br>GTTCCCGCTGTGG-3’	5’-CCACAGCGGGAACCC<br>ATCAGCGATTAG-3’
	CAAT	409-412	5’-CCCGCTGTGGGCCC<br>ATCAGCTTGGGAG-3’	5’-CTCCCAAGCTGATGG<br>GCCCACAGCGGG-3’
	CAAT	378-381	5’-GGGATAGGGGCCCA<br>TGGGAGGCCTC-3’	5’-GAGGCCTCCCATGGG<br>CCCCTATCCC-3’

Supplementary Table S2. Statistical Significance of Protein Areas in Absence of DNA

Dunn’s multiple comparisons test	Significance	Adjusted p-value
GS vs. PF-8 0.5h	****	<0.0001
GS vs. PF-8 1h	****	<0.0001
GS vs. PF-8 2h	ns	>0.9999
GS vs. PF-8 3h	****	<0.0001
GS vs. 100ng	****	<0.0001
GS vs. 200ng	****	<0.0001
GS vs. 400ng	****	<0.0001
GS vs. 800ng	****	<0.0001
GS vs. PF-8 4C	ns	>0.9999
PF-8 0.5h vs. PF-8 1h	****	<0.0001
PF-8 0.5h vs. PF-8 2h	****	<0.0001
PF-8 0.5h vs. PF-8 3h	****	<0.0001
PF-8 0.5h vs. 100ng	****	<0.0001
PF-8 0.5h vs. 200ng	****	<0.0001
PF-8 0.5h vs. 400ng	****	<0.0001
PF-8 0.5h vs. 800ng	****	<0.0001
PF-8 0.5h vs. PF-8 4C	****	<0.0001
PF-8 1h vs. PF-8 2h	ns	0.0942
PF-8 1h vs. PF-8 3h	****	<0.0001
PF-8 1h vs. 100ng	ns	>0.9999
PF-8 1h vs. 200ng	ns	>0.9999
PF-8 1h vs. 400ng	ns	>0.9999
PF-8 1h vs. 800ng	ns	>0.9999
PF-8 1h vs. PF-8 4C	ns	>0.9999
PF-8 2h vs. PF-8 3h	****	<0.0001
PF-8 2h vs. 100ng	***	0.0005
PF-8 2h vs. 200ng	****	<0.0001
PF-8 2h vs. 400ng	ns	0.32
PF-8 2h vs. 800ng	****	<0.0001
PF-8 2h vs. PF-8 4C	ns	>0.9999
PF-8 3h vs. 100ng	****	<0.0001
PF-8 3h vs. 200ng	****	<0.0001
PF-8 3h vs. 400ng	****	<0.0001
PF-8 3h vs. 800ng	****	<0.0001
PF-8 3h vs. PF-8 4C	****	<0.0001
100ng vs. 200ng	ns	>0.9999
100ng vs. 400ng	ns	>0.9999
100ng vs. 800ng	ns	>0.9999
100ng vs. PF-8 4C	ns	0.0953

200ng vs. 400ng	ns	0.9638
200ng vs. 800ng	ns	>0.9999
200ng vs. PF-8 4C	*	0.0289
400ng vs. 800ng	ns	0.623
400ng vs. PF-8 4C	ns	>0.9999
800ng vs. PF-8 4C	*	0.0185

Supplementary Table S3: OriLyt DNA Total Length Measurements

**DNA/Condition**	**Predicted length**	**Measured Average**	**Std. Dev**	**n=DNAs**
Unfixed OriLyt	2414bp	2206.8bp	133.1bp	511
Fixed OriLyt	2414bp	2220.4bp	131.4bp	340
Mutant 1 OriLyt	2414bp	2270.7bp	151.0bp	401
Mutant 2 OriLyt	2414bp	2129.3bp	164.8bp	435
Mutant 3 OriLyt	2414bp	2191.8bp	167.7bp	450
